# Clarifying main nutritional aspects and resting energy expenditure in children with Smith-Magenis syndrome

**DOI:** 10.1007/s00431-024-05715-z

**Published:** 2024-08-20

**Authors:** F. Proli, E. Sforza, A. Faragalli, V. Giorgio, C. Leoni, D. Rigante, E. Kuczynska, C. Veredice, D. Limongelli, A. Zappalà, J. Rosati, M. Pennuto, V. Trevisan, G. Zampino, R. Onesimo

**Affiliations:** 1 Center for Rare Diseases and Birth Defects, Department of Woman and Child Health and Public Health, Fondazione Policlinico Universitario A. Gemelli IRCCS, 00168 Roma, Italy; 2 Università Cattolica del Sacro Cuore, 00168 Rome, Italy; 3 Center of Epidemiology, Biostatistics and Medical Information Technology, Marche Polytechnic University, Ancona, Italy; 4 Department of Biomedical Science and Public Health, Marche Polytechnic University, Ancona, Italy; 5 Pediatric Neurology Unit, Department of Woman and Child Health and Public Health, Fondazione Policlinico Universitario A. Gemelli IRCCS, Rome, Italy; 6 Cellular Reprogramming Unit, Fondazione Casa Sollievo Della Sofferenza IRCCS, San Giovanni Rotondo, Viale Dei Cappuccini, 71013 Foggia, Italy; 7 Department of Biomedical Sciences, University of Padova, Padova, Italy; 8 Veneto Institute of Molecular Medicine (VIMM), Padova, Italy

**Keywords:** Smith-Magenis syndrome, Obesity, Nutrition, Resting energy expenditure, Indirect calorimetry, Pediatric disability

## Abstract

Our study aims to define resting energy expenditure (REE) and describe the main nutritional patterns in a single-center cohort of children with Smith-Magenis syndrome (SMS). REE was calculated using indirect calorimetry. Patients’ metabolic status was assessed by comparing measured REE (mREE) with predictive REE (pREE). Patients also underwent multidisciplinary evaluation, anthropometric measurements and an assessment of average energy intake, using a 3-day food diary, which was reviewed by a specialized dietitian. Twenty-four patients (13 M) were included, the median age was 9 years (IC 95%, 6–14 years), 84% had 17p11.2 deletion, and 16% had RAI1 variants. REE was not reduced in SMS pediatric patients, and the mREE did not differ from the pREE. In patients with RAI1 variants (16%, *n* = 3/24), obesity was more prevalent than those with 17p11.2 deletion (100% vs 38%). Lower proteins intake and higher total energy intake were reported in obese and overweight patients, compared to healthy weight children. No significant difference was found between males and females in energy or macronutrient intake. *Conclusions*: In SMS, the onset of obesity is not explained by REE abnormalities, but dietary factors seem to be crucial. Greater concern should be addressed to patients with RAI1 variants. A better understanding of the molecular mechanisms causing obesity in SMS patients could set the basis for possible future targeted therapies.

**Table:** 

**What is Known:** • *More than 90% of SMS patients after the age of 10 are overweight or obese.*
**What is New:** • *Onset of overweight and obesity in SMS pediatric patients is not explained by abnormal resting energy expenditure.* • *The development of syndrome-specific dietary guidelines for SMS patients should be of utmost relevance and are highly needed.*

**Supplementary Information:**

The online version contains supplementary material available at 10.1007/s00431-024-05715-z.

## Introduction

Smith-Magenis syndrome (SMS) (OMIM #182290) is a genetic disorder characterized by peculiar dysmorphisms, intellectual disability, behavioral abnormalities, sleep disturbance, and childhood-onset abdominal obesity. Moreover, patients may also suffer from seizures, hearing loss, scoliosis, cleft lip and/or palate, renal, ocular, or other congenital anomalies [1, 2]*.* Since the first description of this condition, cardiac anomalies have also been reported in patients with 17p11.2 deletion [3] and confirmed recently [4–6].

The etiology of this syndrome is secondary to retinoic acid-induced 1 (*RAI1*) gene haploinsufficiency. This is mainly caused by a recurrent interstitial microdeletion at the 17p11.2 locus (90% of cases), commonly spanning 3.5 Mb. Less frequently, pathogenic variants in the *RAI1* gene itself are responsible (10% of cases) [7]*.* Patients with *RAI1* variants are more likely to exhibit characteristic behavioral patterns and severe obesity [3, 8]*.*

SMS newborns tend to follow a normal growth pattern, which precedes a deceleration in weight gain during infancy due to feeding difficulties, such as oral motor dysfunction with poor sucking, and swallowing and textural aversion or gastroesophageal reflux disease [2, 9–12]. Nutritional patterns may also be hindered by hypotonia and hyporeflexia [13, 14]*.* During school age and adolescence weight management becomes a concern, with more than 90% of SMS individuals being overweight or obese after the age of 10 [2].

Despite many reports about obesity, our knowledge concerning energy metabolism in pediatric patients with SMS is poor. Resting energy expenditure (REE) represents the amount of calories required for a 24-h period during a non-active period. Its assessment is a valid tool to understand nutritional features in children with rare diseases [15]*.* Indirect calorimetry (IC) is the gold standard for measuring REE [16]. However, in practice, predictive resting energy expenditure (pREE) is often calculated using validated prediction equations based on age, sex, and anthropometrics.

Given the relevance of these aspects, we prospectively performed a quantitative and qualitative description of nutritional intakes in a single-center cohort of SMS pediatric patients. Hence, the aim of this study was to understand the causes of obesity in SMS. A genotype-obesity phenotype correlation of the syndrome was also established.

## Patients and methods

A longitudinal observational study in SMS patients was conducted at the Center for Rare Diseases and Birth Defects, Fondazione Policlinico Universitario A. Gemelli Roma, Italy. All pediatric patients with a clinical and molecular diagnosis of SMS were consecutively enrolled over a period of 3 years (September 2020–September 2023).

The study was conducted according to the Declaration of Helsinki and was approved by the local Ethical Committee as part of a larger study on nutritional aspects in patients with disabilities and rare diseases. Informed consent was obtained from all parents.

Patients were consecutively included in the present study according to the following inclusion criteria: a confirmed genetic diagnosis of SMS, body weight ≥ 10 kg (in line with the device instruction used to assess energy expenditure), age 1 to 18 years. Exclusion criteria considered were absence of genetic confirmation, no compliance to study procedures, significant disease that modify energy expenditure (e.g., cardiovascular or respiratory failure, kidney, liver or inflammatory diseases), and absence of informed consent.

All patients underwent anthropometric measurements, assessment of average energy intake (AEI) and REE evaluation. Anthropometric measurements were performed in triplicate by the same investigator (EK) and included body weight, height, and body mass index (BMI) with criteria established by the World Health Organization (WHO) [17]*.* The weight was evaluated using a digital scale accurate to 0.1 kg. The height was measured in a recumbent (under 2 years old) or standing position, with an accuracy to the nearest 0.1 cm. Weight, height, and BMI were converted to standard deviation (SD) or percentile scores with reference to CDC 2000 data [18]*.*

Dietary intake was assessed using a 3-day food diary, two on weekdays and one at the weekend. Average energy intake (kcal/day), protein intake (g/kg/day, %), carbohydrate intake (%), fat intake (%), and liquid intake (ml/kg) including sweetened beverages were evaluated [19]. Liquid intake was recorded in increments of 100 ml/kg, ensuring consistency and accuracy in the measurements. The diary was carefully explained by an experienced dietician and was recorded by parents and then reviewed by the same specialized dietician. During 3 days, all daily meals and snacks eaten were recorded continuously throughout the day. For each meal, participants were requested to report an exhaustive description of food and recipes, food amount measured using a scale and brand of packaged foods consumed. All diaries were analyzed using an Excel spreadsheet to estimate the composition of the macronutrients of the diet and the frequency of foods. The nutrient composition and energy of food were derived from the Food Composition Database for Epidemiological Studies in Italy (Banca Dati di Composizione degli Alimenti per Studi Epidemiologici in Italia—BDA) [20]. Expected energy and macronutrient intake were defined according to the age- and gender-based and weight-dependent LARN (Livelli di Assunzione di Riferimento di Nutrienti ed energia per la popolazione italiana) by the Italian Society of Human Nutrition (SINU) [21]. The level of physical activity was also registered.

The measured REE (mREE) was determined by IC using an open-circuit calorimeter (QUARK RMR open-circuit indirect calorimeter by Cosmed Italy). Its accuracy and use in syndromic patients have been described in a previous report [15]. The machine was calibrated automatically before each measurement in accordance with the manufacturer’s instructions. Patients lay in a supine position for 30 min with a canopy placed over their heads during the measurement. The first 10 min of the measurements were needed to ensure that the patient was settled and that the air inside the canopy reached a steady state, the following 20 min were used to calculate REE. Fasting for a minimum of 6 h was mandatory in order to record a reliable estimation. The measurements took place in a thermo-neutral environment (ambient temperature 24–26 °C) deprived of any external stimuli. Steady state was determined by five consecutive minutes in which VO2 and VCO2 variations were less than 10%. Averaging the steady state values allowed the determination of 24 h REE, done by using the abbreviated Weir equation: REE Kcal/day = (3.941 VO2 mL/min + 1.106 VCO2 mL/min) × 1.44 [22].

Measured REE values were compared with pREE based on the Schofield, Harris-Benedict, Mifflin, and Muller equations [23–25].

Patients’ metabolic status was classified as hypermetabolic if their percentage pREE (defined as mREE/pREE × 100) was > 110%, hypometabolic if their percent pREE was < 90%, and normal if their percent pREE was between 90 and 110% [26, 27].

### Statistical analysis

Given the small sample size, a non-parametric approach was used. Medians with the interquartile ranges (IQR) and absolute frequencies with percentage summarized quantitative and qualitative variables, respectively. The differences between males and females were investigated with the Wilcoxon sum-rank test and with chi-square or Fisher exact test.

The differences between measured REE and predicted REE calculated with the 3 different equations (Schofield, Harris-Benedict, Mifflin, and Muller) were investigated using the Wilcoxon sum-rank test.

A comparison between measured and expected values of total energy intake, proteins, and liquids was also performed via Wilcoxon sum-rank test.

The Fisher exact test was also used to evaluate the association between BMI classes (underweight, normal weight, overweight, obese) and patient’s metabolic status. A sensitivity analysis compared total energy intake, macronutrients (carbohydrates, lipids, proteins), measured REE, and age between overweight and obese patients versus underweight or healthy weight ones. A *p*-value less than 0.05 was considered statistically significant. The whole analysis was performed using R statistical software, version 4.3.2.

## Results

Among the 28 children with SMS considered eligible, 4 were excluded: 3 due to reduced compliance with the IC and study procedures and 1 due to the presence of a non-pathogenic *RAI1* variant. All data were collected successfully with no missing values across the variables analyzed.

Twenty-four patients were included in the analysis with a median age of 9 years (IQR, 6–14 years): 11 females and 13 males, 21 had 17p11.2 deletion, while 3 had *RAI1* variants (Supplementary Table 1).

No significant differences were detected between males and females in clinical and anthropometric characteristics (Table 1). Eleven patients (46%) were obese (54.5% male), 5 (21%) were overweight (80% males), 7 (28%) had normal weight (57.1% female), and 1 (4%) underweight patient was female.

**Table 1:** Distribution of patients’ characteristics according to the gender

Patients’ characteristics	All (*n* = 24)	Female (*n* = 11)	Male (*n* = 13)	*p*
Age [years, median (IQR)]	9.1 (6.4; 14.04)	9.1 (7.1; 12.66)	9.1 (6.4; 14.6)	0.954^a^
Weight-for-age [SD, median (IQR)]	0.7 (− 0.3; 2.1)	0.5 (− 1.1; 1.6)	0.8 (0.01; 2.2)	0.173^a^
Height-for-age [SD, median (IQR)]	− 0.7 (− 1.3; 0.03)	− 1 (− 1.2; 0.05)	− 0.6 (− 1.8; 0)	0.794 ^a^
BMI [kg/m^2^, median (IQR)]	23.5 (16.8; 27.3)	23.2 (15.65; 25.35)	24.8 (17.8; 29.8)	0.224 ^a^
BMI-for-age [percentile, median (IQR)]	92.6 (74.1; 99)	84 (42.9; 96.5)	93.8 (85; 99)	0.256 ^a^
BMI-for-age [SD, median (IQR)]	1.6 (0.5; 2.2)	0.98 (− 0.28; 1.88)	1.6 (1.03; 2.4)	0.203 ^a^
BSA [m^2^, median (IQR)]	1.2 (0.8; 1.48)	1.2 (0.8; 1.3)	1.2 (0.8; 1.73)	0.464 ^a^
Weight status category [*n* (%)]				0.486^b^
Underweight	1 (4)	1 (9.1)	0 (0)	
Normal weight	7 (29)	4 (36)	3 (23)	
Overweight	5 (21)	1 (9.1)	4 (31)	
Obese	11 (46)	5 (45)	6 (46)	
*RAI* variant [yes, *n* (%)]	3 (13)	2 (18)	1 (7.7)	0.576^b^

*BSA*, body surface area; *p* refers to ^(a)^ Wilcoxon sum-rank test, ^(b)^Fisher exact test; *n*, number

All patients with *RAI1* variants (*n* = 3, 6%) were obese, vs 38% of those with 17p11.2 deletion (14% females and 24% males).

**Fig. 1:**
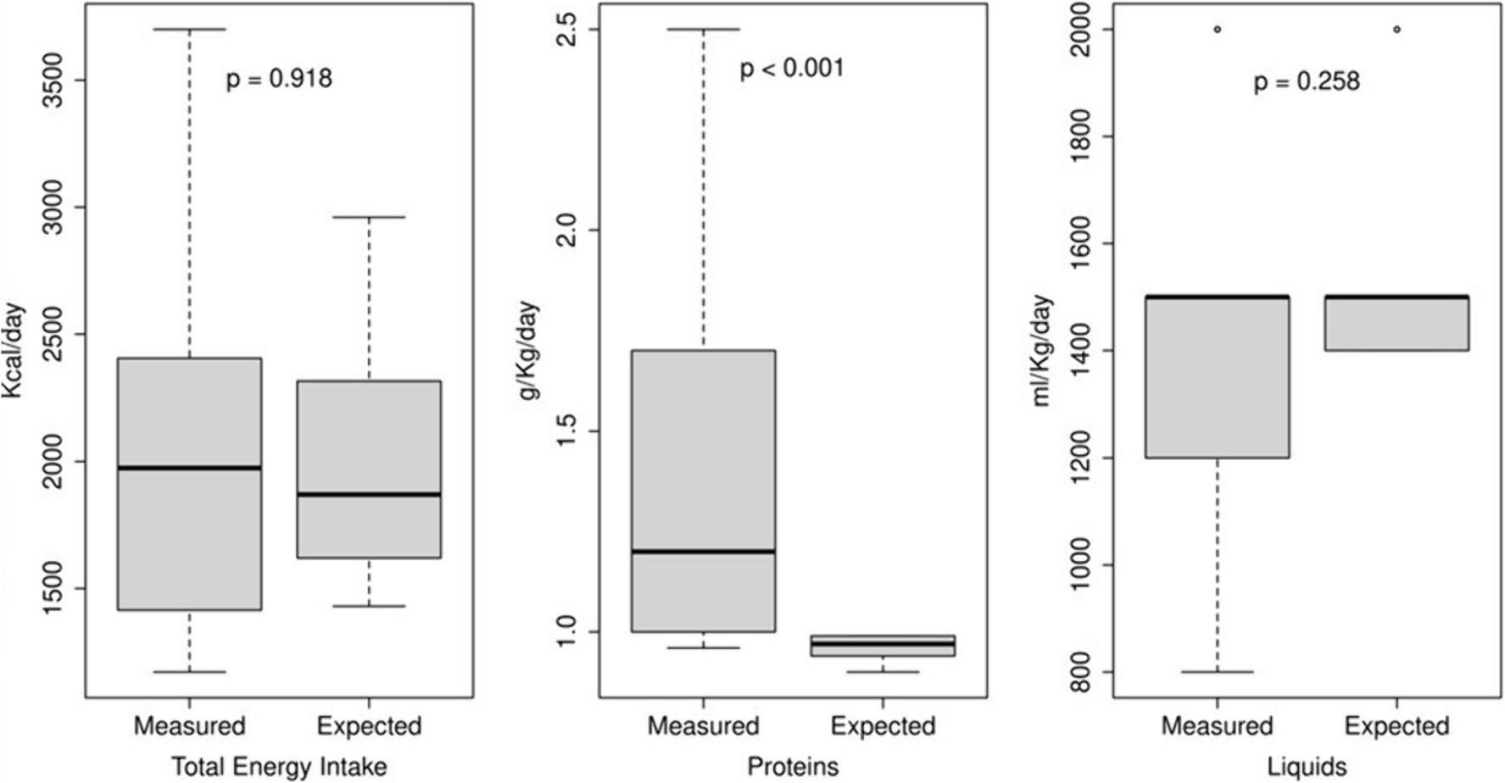
Comparison between measured and expected intakes. Measured total energy intake (kcal/day), proteins intake (g/kg/day), and liquids intake (ml/kg/day) as reported in the 3-day food diary compared with the expected intakes defined according to the age- and gender-based and weight-dependent LARN by the Italian Society of Human Nutrition

Table 2 shows the total daily energy and macronutrients’ intake obtained from parents’ report of the 3-day food diary. No significant difference was found between males and females (*p* > 0.05). Patients reported significantly higher values in measured proteins respect to the expected ones (*p* < 0.001), while no difference was found for the total energy intake and liquids’ intake (Fig. 1). Among obese individuals, sugar-sweetened beverage intake was elevated. All measured values showed higher variability than the expected ones, due to the high age variability among patients.

**Table 2:** Total energy intake and macronutrients’ intake obtained from 3-day food diary

	All (*n* = 24)	Female (*n* = 11)	Male (*n* = 13)	*p*
Total energy intake [Kcal/day. median (IQR)]	1974 (1422; 2402)	2000 (1250; 2300)	1949 (1800; 2410)	0.271
Carbohydrates [% of daily food. median (IQR)]	48 (45; 53.5)	48 (45.1; 53.5)	50 (49.4; 53)	0.336
Lipids [% of daily food. median (IQR)]	35 (33.2; 37)	35 (33.5; 37.7)	35 (33.3; 37)	0.725
Proteins [% of daily food. median (IQR)]	15 (11.9; 15.9)	15.9 (10.5; 16.5)	15 (13; 15)	0.559
Proteins [g/kg. median (IQR)]	1.2 (1; 1.7)	1.7 (1.5; 2)	1.2 (1; 1.2)	0.159
Liquids [ml/kg. median (IQR)]	1500 (1200; 1500)	1300 (1025; 1500)	1500 (1400; 2000)	0.051

*p* refers to the Wilcoxon sum-rank test

*n*, number

Table 3 shows the comparison between mREE and pREE according to the Schofield, Harris-Benedict, and Mifflin and Muller different equations. Measured REE was significantly greater than pREE according to the Mifflin and Muller equation when considering the whole population (both males and females). Measured REE was also often higher than pREE based on Schofield and Harris-Benedict equations, although no significant difference was detected.

**Table 3:** Comparison between measured REE and predicted REE

	Measured	Schofield	*p*	Harris-Benedict	*p*	Mifflin and Muller	*p*
All (*n* = 24)							
[median (IQR)]	1380 (1103; 1613)	1358 (962.8; 1726.3)	0.574^a^	1281.4 (986.9; 1530)	0.212^b^	1137 (861.4; 1442.5)	0.046^c^
Female (*n* = 11)							
[median (IQR)]	1364 (948; 1568)	1309.7 (892.8; 1413.8)	0.300^a^	1299.9 (1010; 1324.1)	0.365^b^	1114 (736.4; 1168.8)	0.116^c^
Male (*n* = 13)							
[median (IQR)]	1393 (1168; 1835)	1364.4 (995.9; 1827.7)	0.840^a^	1220.8 (904.3; 1690)	0.448^b^	1197.6 (912.4; 1577.8)	0.264^c^

*p* refers to the Wilcoxon sum-rank test: ^a^measured REE vs predicted REE according to Schofield equation; ^b^measured REE vs predicted REE according to Harris-Benedict equation; ^c^measured REE vs predicted REE according to Mifflin equation

*n*, number

No association was found between metabolic status (hypometabolic, normometabolic, hypermetabolic) and weight classes based on BMI values (Table 4).

**Table 4:** Association between metabolic status and weight status

	Weight status				
	Underweight, *n* (%)	Normal weight, *n* (%)	Overweight, *n* (%)	Obese, *n* (%)	*p*
Metabolic status					
Schofield					0.457
Hypometabolic	0 (0)	0 (0)	0 (0)	3 (27)	
Normometabolic	1 (100)	2 (29)	3 (60)	4 (36)	
Hypermetabolic	0 (0)	5 (71)	2 (40)	4 (36)	
Harris-Benedict					0.476
Hypometabolic	1 (100)	0 (0)	0 (0)	1 (9.1)	
Normometabolic	0 (0)	3 (43)	3 (60)	5 (45)	
Hypermetabolic	0 (0)	4 (57)	2 (40)	5 (45)	
Mifflin					0.111
Hypometabolic	0 (0)	0 (0)	0 (0)	0 (0)	
Normometabolic	0 (0)	0 (0)	2 (40)	45.45 (50)	
Hypermetabolic	1 (100)	7 (100)	3 (60)	54.54 (50)	

*p* refers to the Fisher exact test

*n*, number

Obese or overweight subjects reported lower percentage of proteins in daily food (*p* = 0.024) in comparison with underweight or healthy weight subjects. No difference was found in the percentage of carbohydrates and lipids. Obese or overweight patients showed significantly higher mREE (*p* = 0.023) and higher total energy intake (*p* = 0.017) than underweight or healthy weight patients (Supplementary Table 2). The ratio between the total energy intake and mREE was higher in obese and overweight patients, although not significantly different (*p* = 0.111).

## Discussion

An accurate assessment of energy requirements and definition of the optimal method of nutrient delivery, including oral, enteral and parenteral route are critical elements in the care of children with disability and rare diseases [28, 29]. Furthermore, a thorough understanding of energy balance is required to develop strategies and reduce syndromic obesity [30].

Previous research studies showed that more than 90% of SMS patients after the age of 10 are overweight or obese, and severe obesity might also lead to increased risk for related health issues (e.g., type 2 diabetes and hypercholesterolemia) in adulthood [2]*.*

In our study, less than 70% of children were overweight or obese with no gender preponderance. However, considering the young age of our cohort (median age of 9 years), the real obesity prevalence might be underestimated and therefore will be reassessed in the future. Indeed, food-related behavioral problems like hyperphagia and food foraging at night, along with a sedentary lifestyle and psychotropic medication side effects (affecting appetite/weight gain), typically deteriorate with school age [2]*.* In our series, overweight was seen only in patients older than 9 years. This strongly suggests that 9–10 years should be considered the critical age threshold for bodyweight gain.

Surprisingly, our data confirmed that higher BMI is positively correlated with age, but in contrast with the previously published study by Alaimo et al. [31], no association with the percentage of carbohydrate or fat intake could be established. This study suggested that *Rai1-*haploinsufficient mice were more susceptible to diet induced obesity, and a high fat or high carbohydrate diet might trigger early onset obesity in SMS patients [31].

On the other hand, in our cohort, obese children showed unbalanced protein consumption in favor of other nutrients than normal weight patients. Even though these findings need to be confirmed in larger controlled studies, this result suggests that potentially high-protein diet might modify obesity prevalence in these patients.

An adequate diet for SMS patients requires the development of personalized nutritional plan in relationship with patients’ age, gender, and nutritional status. Interestingly, dietary guidelines for patients with Prader-Willi syndrome (PWS), a common form of syndromic obesity, are well documented in the literature [32, 33]. Specifically, the distribution of macronutrients is in favor of the protein and complex carbohydrate ratio, with an adequate amount of fiber and a limited fat ratio [32]. However, given the lack of dietary SMS-specific indications and the absence of evidence to support increasing or limiting proteins, vitamins, and minerals in this population in relation to the onset of obesity, diet recommendations should follow the balanced, normocaloric type in line with LARN suggestions [21].

For the first time, to our knowledge, REE using IC and the metabolic status in children with SMS were assessed. Specifically, REE is the most important contributor to the total energy expenditure (TEE), namely the amount of energy that individuals use daily. REE accounts for 50–70% of TEE. The other main contributors to TEE are physical activity, linear growth, and thermic effects of food intake and digestion [15].

In several medical conditions [26, 30, 34–36], REE is routinely estimated using standard equations and guides nutrient delivery in critically ill children. Previous studies [29, 37] concomitantly measured both TEE as well as REE in children with obesity, concluding that reduced REE on its own is not the major cause of common obesity. Abawi et al. showed that mean REE% (ratio between mREE and pREE) was higher in children with non-syndromic genetic obesity and lower in children with hypothalamic obesity compared to multifactorial obesity.

Predictive equations are the main clinical tools for determining REE. However, their precise application in overweight and obese syndromic patients is unclear.

REE was not reduced in SMS pediatric patients. The mREE of children with SMS was well-correlated with pREE (*p* > 0.05) calculated with all validated equations tested, except for Mifflin and Muller equation which was mostly used for adolescents with severe obesity [24]. The different median age of our study population comparing to the one of Steinberg A. et al. (9.1 years vs 15.9 years, respectively) might explain this result. No higher prevalence of hypometabolic status was found in overweight or obese patients: mREE was never significantly lower than pREE.

Taking into consideration all these findings, the higher ratio between energy intake and expenditure in obese patients, compared to normal weight ones, might be explained by the higher energy intake (overfeeding) associated with decreased physical activity rather than slower metabolic rate.

Studies in children with PWS show that their reduced REE can be explained by the reduced fat-free mass associated with the syndrome [38]. Future studies on body composition will probably unravel the multifaceted nature of obesity in SMS patients.

Our data confirm that obesity had a higher prevalence in patients with *RAI1* variants (*n* = 3, 16%) than those with 17p11.2 deletion (100% *vs* 38%). In line with our data, obesity was previously found in 12.9% of individuals with 17p11.2 deletion and in 66.7% of patients with *RAI1* variants [3]*.* Previous studies have tried to correlate obesity and gender with contradictory outcomes. Edelman et al. [3] reported that obesity and eating disorders were more prevalent in the female gender, while, on the contrary, Gandhi et al. found that males with SMS might be more overweight and exhibit more severe eating behaviors than females [11]*.* In our study, 11 males versus 6 females were overweight or obese, but given the gender composition of our study, these results were not considered statistically significant.

The molecular involvement of *RAI1* gene in metabolic homeostasis and how its pathogenetic variants predispose to obesity still need to be defined clearly. It has been hypothesized that *RAI1* positively controls the transcription and the expression of several anorexogenic hormones [39] such as proopiomelanocortin and cholecystokinin. The downregulation of these hormones has been detected by measuring their concentrations in blood samples of *Rai1* ± murine models [39].

Moreover, *Rai1*-mice consume more food and show reduced satiation compared to wild type mice, because of a dysregulated signaling system affecting eating behavior [39]*.*

By combining transcriptomic and lipidomic analyses, Turco et al. found that SMS patients had an altered expression of lipid and lysosomal genes, a deregulation in expression of gene implicated in lipid metabolism, lysosome activity, protein/lipid trafficking, impaired mechanism of autophagy/mitophagy, and increased cellular death with reactive oxygen species production [40].

From the clinical standpoint, nutritional issues could strongly influence other important aspects of the psychological profile of SMS patients, such as sleep disorders [41]. Food behavioral abnormalities increase with age, starting mostly at school age, peaking into a specific feeding disorder/severe overeating issue [42] in the early adolescence. The triggering factors causing transition from infancy to the onset of obesity in early adolescence with food-related problems, including impairment of satiety and impulsive response when food is denied, are still not well understood [9]. Even though a molecular deregulation is considered the common ground for eating behaviors, sleep, and obesity among individuals with SMS, it is highly plausible that other yet unknown biological factors might contribute as well [11].

Some limitations should be declared. Missing the exact amounts of sugar-sweetened beverages and their contribution to total energy intake and/or carbohydrate intake requires that specific further observations are needed. Moreover, body shape or body composition lack of assessment also provides the opportunity for future research to better characterize fat distribution patterns in SMS patients. In the future, further studies on nutritional/hydration status assessment by examining the body composition might collect data regarding energy expenditure in SMS patients. Furthermore, the development of syndrome-specific dietary guidelines for SMS patients, as those created for PWS, might be of relevance aiming to hamper weight gain in this cohort of patients, even is not borne out by the data presented in this study.

Our results emphasize further the importance of a personalized clinical and nutritional management of SMS patients, especially in the pre-adolescent age when the risk of bodyweight gain seems to peak. As mostly the exogenous nutritional factors may play a role in the onset of the obesity in these syndromic children, an early dietary intervention, such by correcting the energy balance between macronutrients (increasing protein intake with a lower carbohydrate/lipid consumption), might reduce the risk of the outbreak of overweight.

## Conclusions

The onset of overweight and obesity in SMS pediatric patients is not explained by REE abnormalities, but dietary factors result crucial. Special attention should be given to patients with *RAI1* variants due to their distinct nutritional and metabolic profile. A better understanding of the molecular mechanisms causing obesity in SMS patients could throw the basis for potential future targeted therapies.

## Supplementary Information

Below is the link to the electronic supplementary material.

Supplementary file1 (DOCX 246 KB)

## Data Availability

No datasets were generated or analysed during the current study.

## Abbreviations

BMI: Body mass index
IC: Indirect calorimetry
mREE: Measured resting energy expenditure
pREE: Predictive resting energy expenditure
*RAI1*: Retinoic acid induced 1
SMS: Smith-Magenis syndrome
