# Supplementary material for: Clarifying main nutritional aspects and resting energy expenditure in children with Smith-Magenis syndrome
Source: Eur J Pediatr. 2024 Aug 20;183(10):4563–71. doi: 10.1007/s00431-024-05715-z (PMC11413128; doi:10.1007/s00431-024-05715-z)
Supplement: Supplementary file 1 — Supplementary file1 (DOCX 246 KB) [file 431_2024_5715_MOESM1_ESM.docx]

**Supplementary Table 1: Genetics findings and BMI absolute values and classification according to Clinical Growth Charts (CDC)**

| Patients | | | Genetics | | | | BMI* | | |
| --- | --- | --- | --- | --- | --- | --- | --- | --- | --- |
| # | **Age (y)** | **Testing technique** | | **17p11.2 deletion size (Mb)** | ***RAI1* sequence variants** | **Absolute value** | | **Classification** |  |
| 1 | 2.5 | CGH array | | 4 |  | 12.9 | | UW |  |
| 2 | 11.5 | FISH | | + |  | 24.5 | | OB |  |
| 3 | 9.6 | CGH array | | 3,6 |  | 21.1 | | OW |  |
| 4 | 10.2 | CGH array | | 3,44 |  | 29.8 | | OB |  |
| 5 | 13.8 | NGS | | / | c.3828delC p.Lys1277Argfs*38 | 33.3 | | OB |  |
| 6 | 4.1 | CGH array | | 3.5 |  | 16.9 | | OB |  |
| 7 | 26 | FISH | | + |  | 37.5 | | OB |  |
| 8 | 14.6 | FISH | | + |  | 40.3 | | OB |  |
| 9 | 7.8 | CGH array | | 2,1 |  | 26.9 | | OB |  |
| 10 | 8.9 | NGS | | / | c.373C>T p.Gln125* | 28.3 | | OB |  |
| 11 | 27.3 | CGH array | | 4 |  | 26.6 | | NW |  |
| 12 | 4.7 | CGH array | | 3,9 |  | 15.1 | | NW |  |
| 13 | 9.1 | NGS | | / | c.3828delC p.Lys1277Argfs*38 | 23.2 | | OB |  |
| 14 | 10.7 | FISH | | + |  | 23.8 | | OB |  |
| 15 | 9.1 | FISH | | + |  | 19.8 | | OW |  |
| 16 | 26.9 | FISH | | + |  | 30.4 | | OB |  |
| 17 | 7.8 | CGH array | | 1,5 |  | 15.1 | | NW |  |
| 18 | 19.4 | CGH array | | 3,3 |  | 19.4 | | NW |  |
| 19 | 6.4 | CGH array | | 3,37 |  | 17.8 | | OW |  |
| 20 | 19.8 | CGH array | | 3,7 |  | 26.2 | | OW |  |
| 21 | 5.6 | CGH array | | 4,35 |  | 15.4 | | NW |  |
| 22 | 14.2 | FISH | | + |  | 24.8 | | OB |  |
| 23 | 6.3 | CGH array | | 4,7 |  | 16.6 | | NW |  |
| 24 | 6.3 | CGH array | | 3,37 |  | 15.9 | | NW |  |

Array Comparative Genomic Hybridization; NW= Normal-weight; OW= overweight; OB= Obese; +=deletion identified by Fluorescent In Situ Hibrydization (FISH); Comparative Genomic Hybridization (array CGH); Next generations sequencing (NGS).

**Supplementary Table 2: Macronutrients’ intakes, measured REE and age of subjects according to the weight status**

|  | Overweight/Obese | Underweight/Nomal weight | p |
| --- | --- | --- | --- |
| Energy intake [% of daily food] |  |  |  |
| Carbohydrates | 51 (49.1; 54.5) | 48 (47.3; 51) | 0.131 |
| Lipids | 35 (34; 37) | 35.1 (33; 37.3) | 0.804 |
| Proteins | 14 (11; 15) | 16 (15; 16.4) | 0.024 |
| measured REE  [Kcal/day, median (IQR)] | 1568 (1355; 1739) | 1005.5 (923.2; 1214.8) | 0.023 |
| Total energy intake [Kcal/day. median (IQR)] | 2125 (1838; 2550) | 1365 (1198; 1987) | 0.017 |
| Ratio between Total energy intake and measured REE [median (IQR)] | 1.53 (1.37; 1.55) | 1.37 (1.25; 1.42) | 0.111 |
| Age [years, median (IQR)] | 9.7 (8.8; 14) | 6.4 (6.1; 10.3) | 0.089 |

REE: resting energy expenditure; p refers to the Wilcoxon sum-rank test
